# The top 100 most cited publications on astrocytes in Alzheimer’s disease from 2000 to 2025: a bibliometric analysis

**DOI:** 10.3389/fnins.2025.1593188

**Published:** 2025-09-11

**Authors:** Qi He, Hong Yu, Xinyao Zhou, Kangyi Yang, Wenjie Xiao, Zirui Gao, Qian He

**Affiliations:** ^1^Department of Human Anatomy, Institute of Basic Medicine, North Sichuan Medical College, Nanchong, China; ^2^Midwifery Major, Nursing College, North Sichuan Medical College, Nanchong, China; ^3^Department of Clinical Medicine, North Sichuan Medical College, Nanchong, China; ^4^Nursing Major, Nursing College, North Sichuan Medical College, Nanchong, China

**Keywords:** Alzheimer’s disease, astrocytes, bibliometric analysis, CiteSpace, VOSviewer

## Abstract

**Background:**

The pathogenesis of Alzheimer’s disease (AD) is closely linked to astrocytes. This study conducts a bibliometric analysis of data from a wide range of literature in this field to enhance the in-depth understanding of this area.

**Methods:**

Publications were retrieved from the 2000–2025 Web of Science Core Collection on January 21, 2025. Bibliometrix-package of R, VOSviewer and CiteSpace were used to visualize the research focus and trends.

**Results:**

The number of citations for the top 100 articles ranged from 208 to 602 citations, with a median of 293 and an average of 331.67 citations per article. The author with the most contributions to this collection was Holtzman David M, who authored 7 papers. Most articles originated in the United States (*n* = 69), while Washington University was the institution with the most cited manuscripts (*n* = 40). The *Journal of Neuroscience* contributed the most publications (*n* = 15), followed by *Nature Neuroscience* (*n* = 7). Co-occurrence of keywords analysis unveiled earlier studies focusing on “messenger RNA,” and “IFN-*γ*,” recent studies concentrated on “mechanisms,” and “activation.” Moreover, keywords burst analysis indicated that the most recent prominent keywords were “Aβ,” “activation” and “association” since 2016.

**Conclusion:**

This is the first bibliometric analysis of the top 100 cited research on astrocytes and AD from 2000 to 2025, underscoring that the United States is a prominent leader in this field. Our analysis highlighted the growing interest in the pathogenesis of astrocytes in AD. Future studies on the mechanisms underlying astrocytes in AD will facilitate further research on new therapeutic approaches.

## Introduction

Alzheimer’s disease (AD), also known as dementia, is a neurodegenerative disease characterized by progressive memory loss and cognitive dysfunction (Angelova and Abramov, 2014). According to the World Alzheimer’s Disease Report (Weidner and Barbarino, 2019), the number of dementia patients worldwide is currently about 50 million, of which about two-thirds suffer from AD. With the aging of the population, it is expected that by 2050, the number of dementia patients will increase to 150 million, of which the number of people with AD will increase to 100 million accordingly. One new case of AD is expected to occur every 3 s, resulting in nearly 1 million new cases annually. AD has become the fourth leading killer of older adults after heart disease, cancer, and stroke. During 2000–2017, mortality rates for stroke and heart disease declined, while mortality rates for AD increased by 145% (Alzheimer’s Association Report, 2024; Alzheimer’s Association Report, 2023). The etiology and pathogenesis of AD are very complex, mainly including intracellular neurofibrillary tangles (NFT) and extracellular amyloid-*β* (Aβ) aggregates that form age spots (Shankar et al., 2008; Li et al., 2007). The amyloid cascade hypothesis suggests that Aβ deposition triggers a series of cascading reactions that lead to neuronal degeneration, which is the main reason why cognitive dysfunction occurs in AD (Carter et al., 2019). Glial cells in the central nervous system (CNS) that maintain homeostasis, such as astrocytes, oligodendrocytes, NG2 glial cells, and microglia, etc. are involved in this process; especially, the largest and most numerous astrocytes (Carter et al., 2019; Heneka et al., 2010).

Astrocytes are the most widely distributed, numerous, and largest class of glial cells in the central nervous system. Astrocytes not only support, guide and separate nerve cells (Robel and Sontheimer, 2016; López-Hidalgo and Schummers, 2014), but also have many more complex regulatory functions, participating in neurotransmitter secretion and recycling, secretion of neurotrophic factors and cytokines, promotion of neighboring neuron dendritic development and synaptic connectivity, participation in the internal immune response, transmission of nutrients, and transmission of electrical signals (Anderson et al., 2016; Halassa and Haydon, 2010). Studies have shown that in the brain tissue of patients with AD, Parkinson’s disease, and other diseases, there are a large number of reactive astrocytes in an abnormal state, which are capable of destroying neurons and are the culprits of neurodegenerative diseases (Araújo et al., 2022; Habib et al., 2020). Genetic studies have shown that the overall risk of AD is primarily associated with gene expression in glial cells, with clusterin/apolipoprotein J (CLU/ApoJ), sortilin-related receptor (SORL1), and the fermitin family member 2 (FERMT2) as the main genes involved. SORL1, FERMT2, and apolipoprotein E (ApoE4) (Balcar et al., 2021; Mishra et al., 2022; Sullivan et al., 2019), are mainly expressed by astrocytes, and astrocytes undergo a series of morphological, molecular, and functional changes during the course of AD, suggesting that astrocytes play an important role in the pathogenesis of AD. Besides, some researchers have also treated it by inhibiting the production of reactive astrocytes that release neurotoxins. This therapeutic approach is not only effective for AD but also has therapeutic implications for other neurodegenerative diseases.

Currently, despite the abundance of literature on astrocytes in AD, there is a notable lack of comprehensive overview information on the number of relevant publications, countries, authors, institutions, journals, and keywords commonly used in related research. The lack of information makes it difficult to identify research hotspots and emerging research directions in the field. Bibliometrics, as a comprehensive method of quantitative and qualitative analysis, can provide valuable insights into the characteristics of publications (Donthu et al., 2021). The utilization of scientific databases facilitates bibliometric research (Hao et al., 2018). In recent years, bibliometrics has become a popular method for analyzing progress in the field of neurology (Wilson et al., 2021). For example, Zhang et al. (2024a) opted for bibliometric analysis to comprehensively summarize the advancements in the study of microglia in AD. Yang et al. (2024) conducted a bibliometric analysis to identify additional potential biomarkers in AD. Alcibíades et al. (2024) conducted a bibliometric analysis in 2024, while we undertook a similar investigation to gain a more comprehensive understanding of the influential studies concerning astrocytes in relation to AD. The purpose of the present study was to identify the 100 top most-cited publications in astrocytes and AD to highlight the most significant advances in the field over the past several decades. This knowledge can be used to better understand the classical studies that have significantly contributed to the field of astrocytes and AD.

## Materials and methods

### Data source and collection

**Figure 1:**
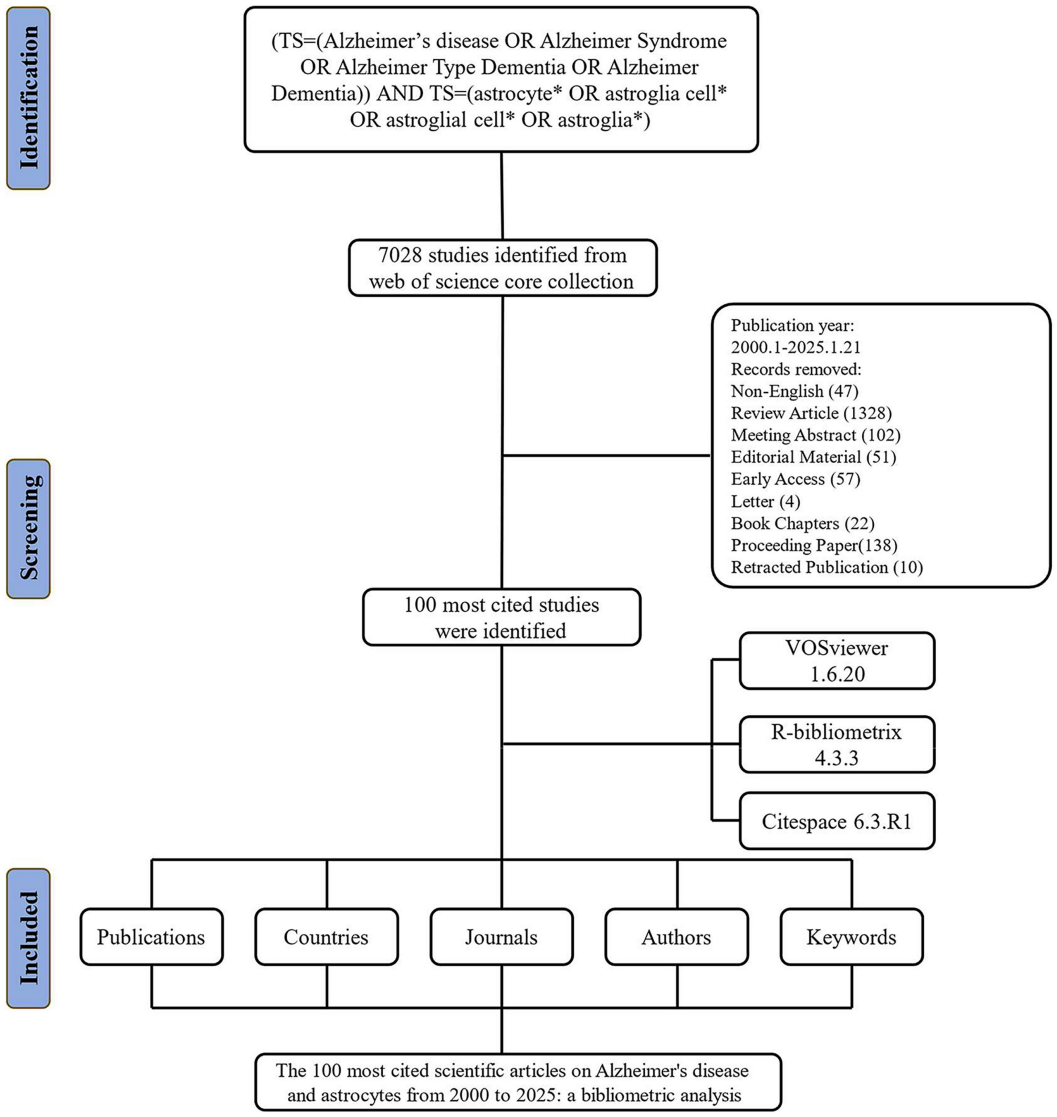
Flow diagram of the bibliographic retrieval process.

The data for this study were extracted from the Science Citation Index Expanded (SCI-EXPANDED) within the Clarivate Analytics Web of Science Core Collection (WoSCC). Although both WoSCC and Scopus can be used for bibliometric analysis, we selected WoSCC because it is a collection of high-quality, globally peer-reviewed academic publications that focuses primarily on traditional academic literature, including journal articles, conference proceedings, and books(Ai et al., 2023). Besides, the WoSCC database is a multidisciplinary and comprehensive database with a complete citation network, providing key bibliometric indices (i.e., JCR, IF, and H-index) (Tian and Chen, 2024). Therefore, we selected it to obtain global academic information for bibliometric analysis according to previous studies (Zhang et al., 2024b; Ai et al., 2024; Wu et al., 2022). In the present study, the search terms were as follows: TS = (Alzheimer’s disease OR Alzheimer Syndrome OR Alzheimer Type Dementia OR Alzheimer Dementia) AND TS = (astrocytes* OR astroglia cell* OR astroglial cell* OR astroglia*) was used to search for relevant articles (An et al., 2024). Document retrieval and recording were concluded on January 21, 2025, to prevent possible bias resulting from subsequent database updates. The inclusion criteria for this study were: (1) Studies published between January 2000 and January 2025; (2) Studies classified as “original articles” in the English language; (3) The top 100 most cited publications. The exclusion criteria were: (1) Exclusion of letters, meeting abstracts, conference proceedings, editorial materials, early access publications, and other non-article or review literature; (2) Exclusion of literature not relevant to the research topic. For each article, two researchers separately screened the title, abstract, and document type. If necessary, the researchers perused the full article for a more comprehensive evaluation of whether to include it in the analysis. The records of the 100 most influential publications were obtained from WoSCC in the “Full Record and Cited References” (Figure 1).

### Data analysis

Bibliometric analyses were conducted utilizing VOSviewer (version 1.6.20), CiteSpace (version 6.3. R1), and R package “Bibliometrix” (version 4.3.3). The Bibliometrix R package is mainly for quantitative analysis (Aria and Cuccurullo, 2017). In Bibliometrix, extraction methods are: authors from the AU field (institutions from AU_UN field and countries from AU_CO field); year of publication from the PY field; keywords from the DE field; citations from the TC field. The functions of Bibliometrix version 4.3.3 in this review were to count the number of publications and their citations, calculate the frequency of used keywords, compute the strength of collaboration among countries/authors, and create a three-field plot of the relationships among countries, institutions, and authors.

VOSviewer (version 1.6.20) is a bibliometric analysis software that can extract key information from numerous publications, which is often used to build collaboration, co-citation, and co-occurrence networks (Wu et al., 2022). VOSviewer calculated the number of publications, citations, and keyword frequency (van Eck and Waltman, 2010). Co-occurrence networks of important keywords in the scientific literature were constructed and visualized using the software’s embedded clustering algorithm. Co-authorship and co-occurrence analyses were the primary focus of this study. VOSviewer was also used to analyze the collaboration of countries, institutions, and authors. The size of nodes represents the number of publications, the thickness of lines symbolizes the strength of the link, and the color of nodes stands for different clusters or times.

CiteSpace, a Java application, is developed by Dr. Chaomei Chen, which allows the detection and visualization of trends and patterns in publications obtained from bibliometric databases (Wang et al., 2024). In our study, CiteSpace was applied to analyze keywords with Citation Bursts (Chen, 2006), setting parameters for time slicing from January 2003 to December 2024. The time slice was set to 1 year; node types: keywords. When the node type was keywords, the threshold (top *N* in each slice) = 5, pruning = pathfinder + pruning merged networks.

Our study employed the h-index to quantify the academic impact of individuals and journals, respectively. The h-index is a vital indication to evaluate the academic contribution of researchers and could predict their future scientific achievements (Bertoli-Barsotti and Lando, 2017; Hirsch, 2005). In this study, the h-index of each author was obtained from WoSCC. The g-index refers to the highest number of papers that receive h-index or more citations (Abbas, 2012). The m-index, defined as (h-index) / (number of years since the author’s first published paper), characterizes the rise in the h-index over time.

## Results

### An overview of publications and publication trend analysis

**Figure 2:**
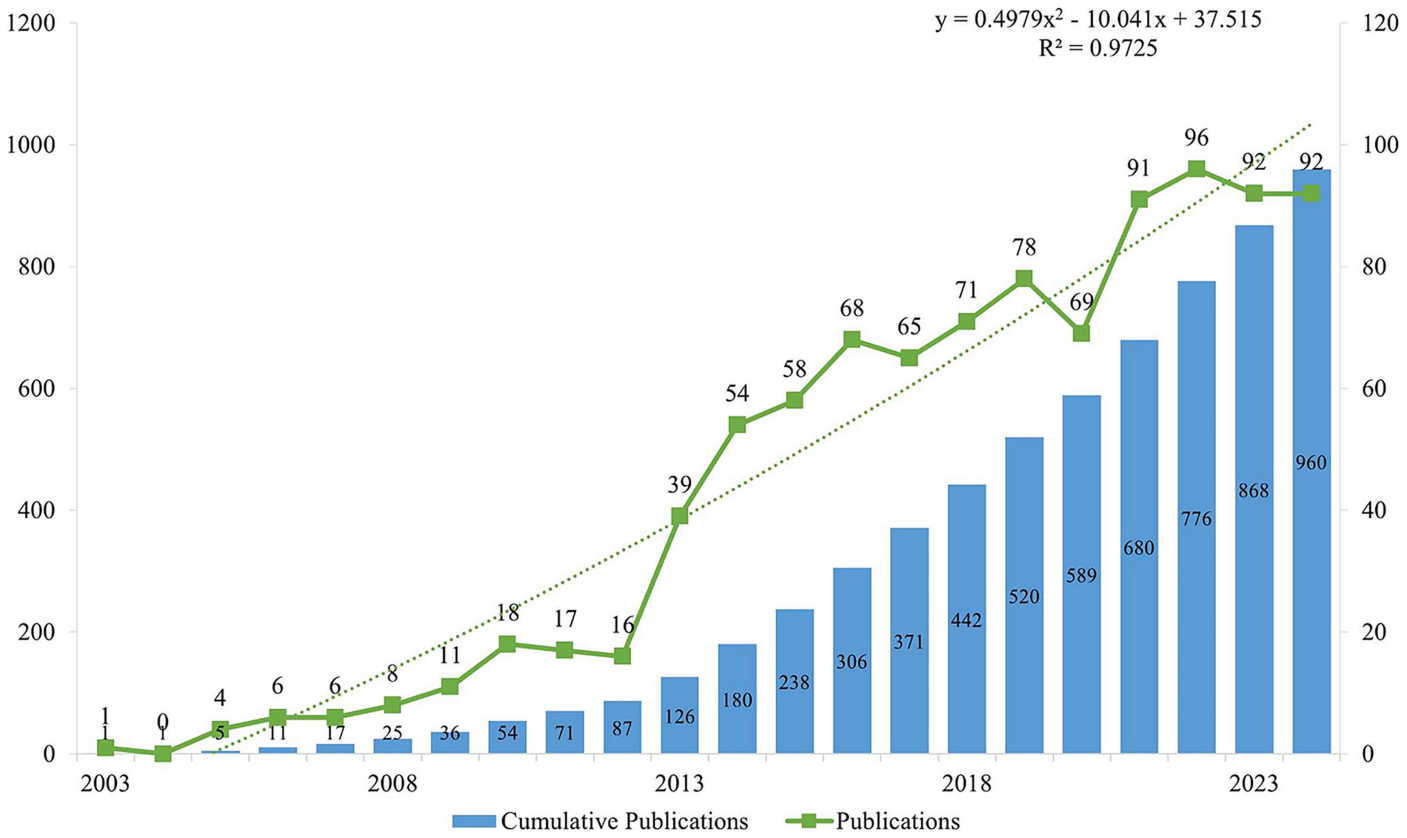
Number of publications per year and the cumulative number.

All the 100 most cited studies were published between 2000 and 2021 (Figure 2). Detailed information on the 100 most influential publications were shown in Table 1. The top 100 articles garnered between 208 and 602 citations, with a median of 292 and an average of 331.67 citations per article. Focusing on the top three studies, the most cited article (“Defects in IGF-1 receptor, insulin receptor and IRS-1/2 in Alzheimer’s disease indicate possible resistance to IGF-1 and insulin signalling”) was published in the *Neurobiology of Aging* in 2010 and was cited 602 times(Moloney et al., 2010). The second most cited paper (“*In vivo* direct reprogramming of reactive glial cells into functional neurons after brain injury and in an Alzheimer’s disease model”) was published in *Cell Stem Cell* in 2014 and received 599 citations (Guo et al., 2014). The third most cited paper titled, “Human and mouse single-nucleus transcriptomics reveal TREM2-dependent and TREM2-independent cellular responses in Alzheimer’s disease,” was published in 2020 in *Nature Medicine* and received 597 citations (Zhou et al., 2020). Earlier publications had an advantage in terms of the total citation count. However, when ranked according to average citations per year, some later publications were found to have a greater impact. For instance, although the above-mentioned article “Human and mouse single-nucleus transcriptomics reveal TREM2-dependent and TREM2-independent cellular responses in Alzheimer’s disease” ranked third for total citations (Zhou et al., 2020), it ranked first for the average number of citations per year with 99.5 (Table 1).

**Table 1:** The 100 most-cited articles on astrocytes in AD.

Paper	DOI	Total Citations	TC per Year	Normalized TC
Moloney Am, 2010, Neurobiol Aging	10.1016/j.neurobiolaging.2008.04.002	602	37.63	1.84
Guo Zy, 2014, Cell Stem Cell	10.1016/j.stem.2013.12.001	599	49.92	1.54
Zhou Yy, 2020, Nat Med	10.1038/s41591-019-0695-9	597	99.5	1.4
Lin Yt, 2018, Neuron	10.1016/j.neuron.2018.05.008	595	74.38	1.61
Johnson Ecb, 2020, Nat Med	10.1038/s41591-020-0815-6	566	94.33	1.33
Jo S, 2014, Nat Med	10.1038/nm.3639	565	47.08	1.46
Kondo T, 2013, Cell Stem Cell	10.1016/j.stem.2013.01.009	561	43.15	1.73
Kuchibhotla Kv, 2009, Science	10.1126/science.1169096	561	33	1.52
Combs Ck, 2000, J Neurosci	10.1523/JNEUROSCI.20-02-00558.2000	543	20.88	1.78
Grubman A, 2019, Nat Neurosci	10.1038/s41593-019-0539-4	530	75.71	1.18
Benito C, 2003, J Neurosci	NA	515	22.39	1.33
Habib N, 2020, Nat Neurosci	10.1038/s41593-020-0624-8	507	84.5	1.19
Spangenberg Ee, 2016, Brain	10.1093/brain/aww016	487	48.7	1.39
Winkler Ea, 2015, Nat Neurosci	10.1038/nn.3966	480	43.64	1.51
Abramov Ay, 2004, J Neurosci	10.1523/JNEUROSCI.4042-03.2004	480	21.82	1.36
Falcon B, 2019, Nature	10.1038/s41586-019-1026-5	474	67.71	1.05
Minter Mr., 2016, Sci Rep-Uk	10.1038/srep30028	466	46.6	1.33
Lian H, 2015, Neuron	10.1016/j.neuron.2014.11.018	461	41.91	1.45
Bhat R, 2012, Plos One	10.1371/journal.pone.0045069	415	29.64	1.31
Martorell Aj, 2019, Cell	10.1016/j.cell.2019.02.014	414	59.14	0.92
Verghese Pb, 2013, P Natl Acad Sci Usa	10.1073/pnas.1220484110	414	31.85	1.28
Park J, 2018, Nat Neurosci	10.1038/s41593-018-0175-4	410	51.25	1.11
Lian H, 2016, J Neurosci	10.1523/JNEUROSCI.2117-15.2016	402	40.2	1.14
Olabarria M, 2010, Glia	10.1002/glia.20967	392	24.5	1.2
Shi Qq, 2017, Sci Transl Med	10.1126/scitranslmed.aaf6295	388	43.11	1.33
Nott A, 2019, Science	10.1126/science.aay0793	381	54.43	0.85
Wahrle Se, 2004, J Biol Chem	10.1074/jbc.M407963200	372	16.91	1.05
Craig-Schapiro R, 2010, Biol Psychiat	10.1016/j.biopsych.2010.08.025	370	23.13	1.13
Zhang Y, 2002, J Cell Biol	10.1083/jcb.200110119	369	15.38	1.15
Park Mw, 2021, Redox Biol	10.1016/j.redox.2021.101947	367	73.4	1.28
Serrano-Pozo A, 2011, Am J Pathol	10.1016/j.ajpath.2011.05.047	362	24.13	1.23
Cosenza-Nashat M, 2009, Neuropath Appl Neuro	10.1111/j.1365-2990.2008.01006.x	356	20.94	0.96
Kisler K, 2017, Nat Neurosci	10.1038/nn.4489	352	39.11	1.21
Nagele Rg, 2003, Brain Res	10.1016/S0006-8993(03)02361-8	352	15.3	0.91
Xu Zq, 2015, Mol Neurodegener	10.1186/s13024-015-0056-1	333	30.27	1.04
Yan Jj, 2001, Brit J Pharmacol	10.1038/sj.bjp.0704047	331	13.24	1.26
Tomiyama T, 2010, J Neurosci	10.1523/JNEUROSCI.5825-09.2010	330	20.63	1.01
Brecht Wj, 2004, J Neurosci	10.1523/JNEUROSCI.4315-03.2004	327	14.86	0.93
Lue Lf, 2001, Glia	10.1002/glia.1072	325	13	1.23
Mostafavi S, 2018, Nat Neurosci	10.1038/s41593-018-0154-9	319	39.88	0.86
Wahrle Se, 2008, J Clin Invest	10.1172/JCI33622	315	17.5	1.17
Mandrekar-Colucci S, 2012, J Neurosci	10.1523/JNEUROSCI.5268-11.2012	313	22.36	0.99
Akama Kt, 2000, J Biol Chem	10.1074/jbc.275.11.7918	311	11.96	1.02
Hawkes Ca, 2009, P Natl Acad Sci Usa	10.1073/pnas.0805453106	310	18.24	0.84
Yamamoto M, 2007, Am J Pathol	10.2353/ajpath.2007.060378	308	16.21	1
Yin Kj, 2006, J Neurosci	10.1523/JNEUROSCI.2085-06.2006	306	15.3	1
Busciglio J, 2002, Neuron	10.1016/S0896-6273(02)00604-9	305	12.71	0.95
Orellana Ja, 2011, J Neurochem	10.1111/j.1471-4159.2011.07210.x	300	20	1.02
Seyfried Nt, 2017, Cell Syst	10.1016/j.cels.2016.11.006	294	32.67	1.01
Simpson Je, 2010, Neurobiol Aging	10.1016/j.neurobiolaging.2008.05.015	294	18.38	0.9
Husemann J, 2002, Glia	10.1002/glia.10148	292	12.17	0.91
Garwood Cj, 2011, Cell Death Dis	10.1038/cddis.2011.50	291	19.4	0.99
Abramov Ay, 2003, J Neurosci	NA	291	12.65	0.75
Freeman L, 2017, J Exp Med	10.1084/jem.20150237	290	32.22	0.99
Kannan S, 2012, Sci Transl Med	10.1126/scitranslmed.3003162	290	20.71	0.92
Phatnani H, 2015, Csh Perspect Biol	10.1101/cshperspect.a020628	286	26	0.9
Orre M, 2014, Neurobiol Aging	10.1016/j.neurobiolaging.2014.06.004	286	23.83	0.74
Goetzl Ej, 2016, Faseb J	10.1096/fj.201600756R	283	28.3	0.81
Riddell Dr., 2008, J Neurosci	10.1523/JNEUROSCI.1972-08.2008	283	15.72	1.05
Zhao J, 2011, J Neuroinflamm	10.1186/1742-2094-8-150	282	18.8	0.96
Urrutia P, 2013, J Neurochem	10.1111/jnc.12244	281	21.62	0.87
Guillemin Gj, 2005, Neuropath Appl Neuro	10.1111/j.1365-2990.2005.00655.x	268	12.76	1
Ou Zr, 2018, Brain Behav Immun	10.1016/j.bbi.2017.12.009	267	33.38	0.72
Narasimhan S, 2017, J Neurosci	10.1523/JNEUROSCI.1230-17.2017	262	29.11	0.9
Coppieters N, 2014, Neurobiol Aging	10.1016/j.neurobiolaging.2013.11.031	262	21.83	0.68
Goetzl Ej, 2018, Ann Neurol	10.1002/ana.25172	261	32.63	0.7
Apelt J, 2001, Brain Res	10.1016/S0006-8993(00)03176-0	260	10.4	0.99
Toledo Em, 2010, Mol Psychiatr	10.1038/mp.2009.72	255	15.94	0.78
Hou Yj, 2021, P Natl Acad Sci Usa	10.1073/pnas.2011226118	254	50.8	0.89
Fang F, 2010, Faseb J	10.1096/fj.09-139634	254	15.88	0.78
Lee Sg, 2008, J Biol Chem	10.1074/jbc.M707697200	254	14.11	0.95
Srinivasan K, 2016, Nat Commun	10.1038/ncomms11295	253	25.3	0.72
Abdul Hm, 2009, J Neurosci	10.1523/JNEUROSCI.1064-09.2009	253	14.88	0.68
Matsuoka Y, 2001, Am J Pathol	10.1016/S0002-9440(10)64085-0	253	10.12	0.96
Gyure Ka, 2001, Arch Pathol Lab Med	NA	250	10	0.95
Furman Jl, 2012, J Neurosci	10.1523/JNEUROSCI.2323-12.2012	247	17.64	0.78
Jordao Jf, 2013, Exp Neurol	10.1016/j.expneurol.2013.05.008	243	18.69	0.75
Neumann H, 2001, Glia	10.1002/glia.1108	239	9.56	0.91
Harkany T, 2000, Eur J Neurosci	10.1046/j.1460-9568.2000.00164.x	238	9.15	0.78
Leng K, 2021, Nat Neurosci	10.1038/s41593-020-00764-7	236	47.2	0.83
Lau Sf, 2020, P Natl Acad Sci Usa	10.1073/pnas.2008762117	235	39.17	0.55
Kraft Aw, 2013, Faseb J	10.1096/fj.12-208660	235	18.08	0.73
Brown J, 2004, J Biol Chem	10.1074/jbc.M402324200	232	10.55	0.66
Kirkley Ks, 2017, J Neuroinflamm	10.1186/s12974-017-0871-0	231	25.67	0.79
Macvicar Ba, 2015, Csh Perspect Biol	10.1101/cshperspect.a020388	231	21	0.72
Li Cy, 2011, Curr Alzheimer Res	NA	231	15.4	0.79
Sasaki N, 2001, Brain Res	10.1016/S0006-8993(00)03075-4	228	9.12	0.87
Delekate A, 2014, Nat Commun	10.1038/ncomms6422	227	18.92	0.59
Oksanen M, 2017, Stem Cell Rep	10.1016/j.stemcr.2017.10.016	225	25	0.77
Cui Jg, 2010, J Biol Chem	10.1074/jbc.M110.178848	224	14	0.69
Zhang Fj, 2015, Neuropsych Dis Treat	10.2147/NDT.S75546	222	20.18	0.7
Hur Jy, 2020, Nature	10.1038/s41586-020-2681-2	221	36.83	0.52
Jin Jj, 2008, J Neuroinflamm	10.1186/1742-2094-5-23	221	12.28	0.82
Hoozemans Jjm, 2001, Acta Neuropathol	10.1007/s004010000251	220	8.8	0.84
Blasko I, 2000, Neurobiol Dis	10.1006/nbdi.2000.0321	220	8.46	0.72
Ben Haim L, 2015, J Neurosci	10.1523/JNEUROSCI.3516-14.2015	219	19.91	0.69
Allaman I, 2010, J Neurosci	10.1523/JNEUROSCI.5098-09.2010	218	13.63	0.67
Rodriguez-Vieitez E, 2016, Brain	10.1093/brain/awv404	216	21.6	0.62
Xia Mq, 2000, J Neuroimmunol	10.1016/S0165-5728(00)00285-X	213	8.19	0.7
Hutchison Er, 2013, Glia	10.1002/glia.22483	208	16	0.64

TC, total citation.

### Analysis of the countries/regions

**Figure 3:**
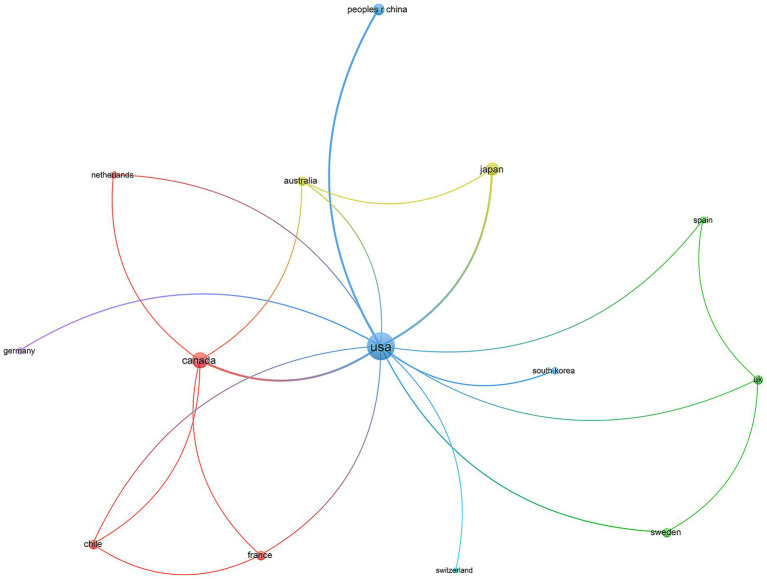
Visualization networks of countries. The collaborative relationships between countries, with nodes representing countries, the size of nodes indicating publication count, and the thickness of links showing the strength of co-authorship collaborations.

As shown in Figure 3, a total of 29 countries/regions contributed to the 100 most cited papers, with 14 of these countries/ regions contributing more than 2 articles. The United States had the highest number of collaborations with other countries, with a total link strength of 28, followed by Canada (total link strength = 9) and Japan (total link strength = 6) (Table 2). The United States played a prominent role in this area relative to other countries and had extensive cooperation with them.

**Table 2:** Publication and citation profiles of leading countries.

Id	Country	Articles	Citations	Total link strength
29	United States	69	24,075	28
4	Canada	8	2,468	9
16	Japan	7	2,769	6
20	China	9	2,454	5
1	Australia	3	1,117	3
5	Chile	3	836	3
9	France	2	519	3
26	Sweden	3	676	3
28	Uk	7	2,438	3
10	Germany	5	1,287	2
17	Netherlands	6	1947	2
24	South Korea	4	1858	2
25	Spain	2	907	2
27	Switzerland	2	471	1

### Analysis of the institutions

**Figure 4:**
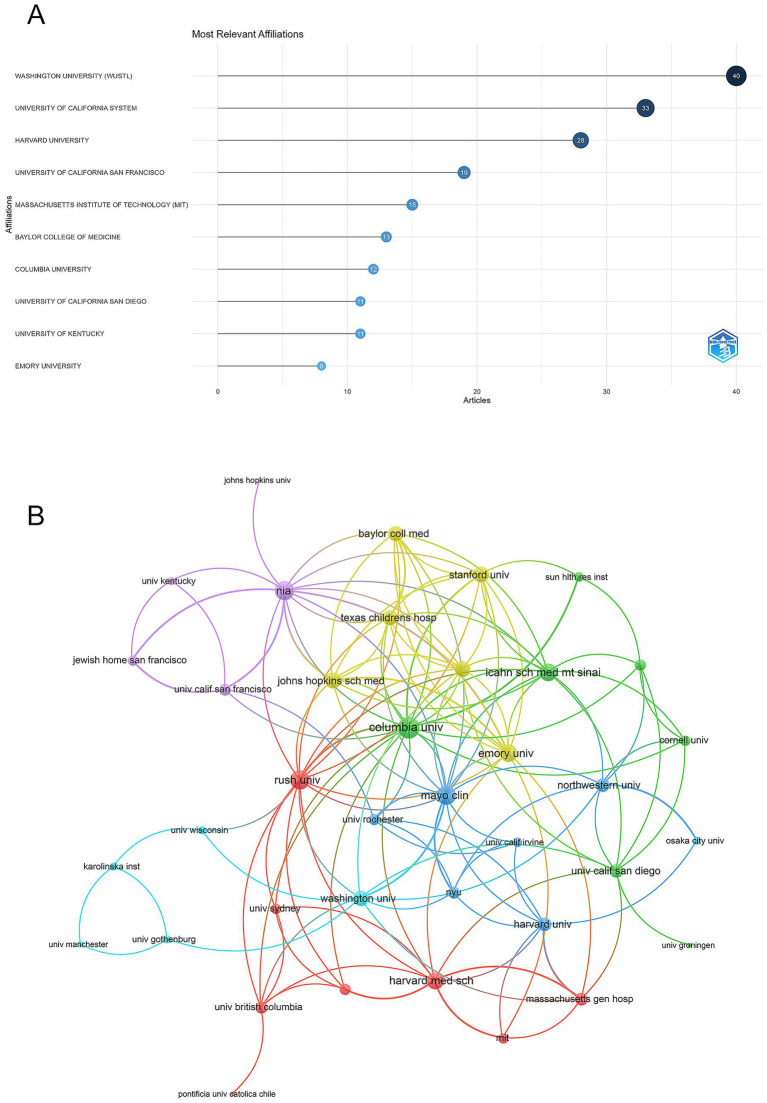
Analysis of institutions. **(A)** Top ten institutions by article count and rank. The circle size shows the article count, with darker shades indicating higher ranks. **(B)** Visualization networks of institution collaborations. Nodes represent institutions, with size indicating publication count. Links represent co-authorships, with thickness showing collaboration strength. Colors indicate different research clusters. Total link strength in collaboration networks measures the frequency of co-authorship between institutions, indicating the level of collaborative research.

A total of 485 institutions contributed to the top 100 cited articles on astrocytes in AD. Washington University (WUSTL) led in the number of publications (*n* = 40), followed by the University of California System (*n* = 33), and Harvard University (*n* = 28) (Figure 4A).

Among the 45 institutions involved in international collaborations with a minimum of 2 articles, Emory University had the highest number of collaborations with other institutions (total link strength = 40), followed by Johns Hopkins University (total link strength = 40) and the University of Texas MD Anderson Cancer Center (total link strength = 38) (Figure 4B).

### Analysis of the authors

**Figure 5:**
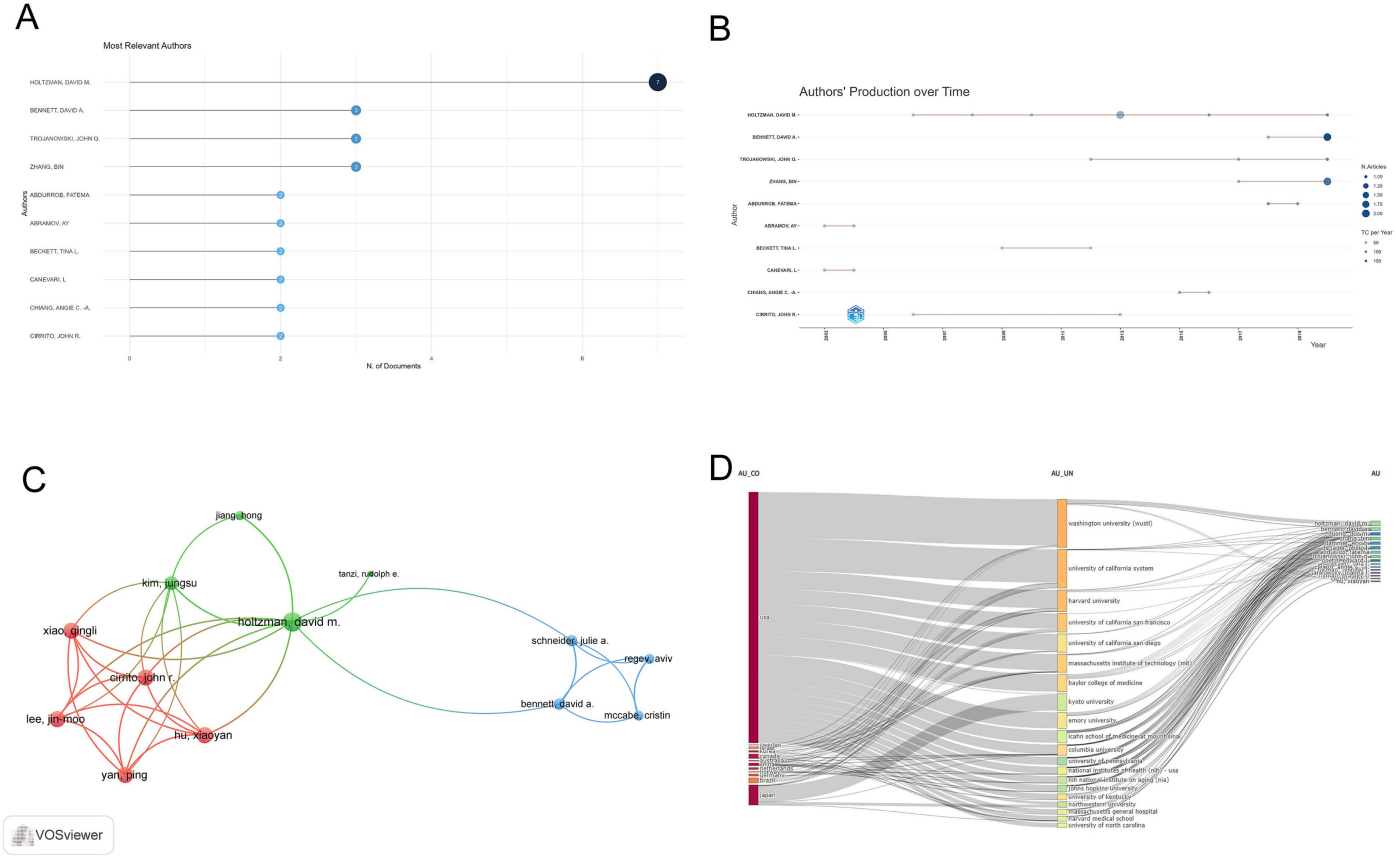
Analysis of authors. **(A)** Top ten authors by article count and rank. The circle size shows the article count, with darker shades indicating higher ranks. **(B)** Top ten authors’ production over time. The number of articles published in a year is indicated by the size of the bubble. The color intensity is proportional to the number of times articles published in that year have been cited. The line represents an author’s publication timeline. **(C)** Visualization networks of author collaborations. Nodes represent authors, with size indicating publication count. Links represent co-authorships, with thickness showing collaboration strength. Colors indicate different research clusters. Total link strength in collaboration networks measures the frequency of co-authorship between authors, indicating the level of collaborative research. **(D)** Three-field plot of the relationships among countries, institutions, and authors. (AU, Author; Au_CO, Author country; AU_UN, Author institutions).

The most relevant authors were identified by employing the “bibliometrix” R package. As demonstrated in Figure 5A, Holtzman David M had the highest number of publications (*n* = 7). Regarding total citations, Holtzman David M. also led the total citations with 2,703 citations, followed by Bennett David A. (1,482) and Trojanowski John Q (1,243) (Table 3). The timeline of publications for the 10 most influential authors was depicted in Figure 5B. The size of each circle corresponds to the number of articles published, with a positive correlation. Additionally, among the 40 authors involved in international collaborations with a minimum of 2 articles, Holtzman David M had the highest number of collaborations with other authors (total link strength = 17), followed by Cirrito John R (total link strength = 11) and Kim Jungsu (total link strength = 8) (Figure 5C). The relationships among countries, institutions, and authors were shown in Figure 5D, with authors from the United States having a substantial impact in this respect.

**Table 3:** Publication and citation profiles of high-impact authors.

Authors	h_index	g-index	m-index	PY_start	TP	TP_Frac	TP_rank	TC	TC_rank
Holtzman David M.	7	7	0.35	2006	7	0.50	1	2,703	1
Bennett David A.	3	3	0.38	2018	3	0.12	2	1,482	2
Trojanowski John Q.	3	3	0.21	2012	3	0.22	3	1,243	3
Zhang Bin	3	3	0.33	2017	3	0.15	4	1,049	4
Abdurrob Fatema	2	2	0.25	2018	2	0.11	5	1,009	5
Abramov Ay	2	2	0.09	2003	2	0.67	6	771	29
Beckett Tina L.	2	2	0.12	2009	2	0.21	7	500	44
Canevari L	2	2	0.09	2003	2	0.67	8	771	29
Chiang Angie C. -A.	2	2	0.18	2015	2	0.25	9	863	14
Dammer Eric B.	2	2	0.22	2017	2	0.13	10	860	17
Duchen Mr	2	2	0.09	2003	2	0.08	12	771	29
Duong Duc M.	2	2	0.22	2017	2	0.67	13	860	17
Eikelenboom P	2	2	0.08	2000	2	0.09	14	440	49
Furman Jennifer L	2	2	0.12	2009	2	NA	NA	500	44
Hu Xiaoyan	2	2	0.10	2006	2	0.21	16	541	39
Hyman Bradley T.	2	2	0.12	2009	2	0.33	17	923	8
Jankowsky Joanna L.	2	2	0.18	2015	2	NA	NA	863	14
Kapogiannis Dimitrios	2	2	0.11	2008	2	0.39	19	729	32
Kim Jungsu	2	2	0.20	2016	2	0.25	20	544	36
Klein William L.	2	2	0.11	2008	2	0.19	21	550	35

H_index, The h-index of the author, which measures both the productivity and citation impact of the publications; g_index, The g-index of the author, which gives more weight to highly-cited articles; m_index, The m-index of the author, which is the h-index divided by the number of years since the first published paper. TP, Total Publications; TP_Frac, Fraction of Total Publications; TP_rank, Rank of Total Publications; TC, Total Citations; TC_rank, Rank of Total Citations; PY_start, Publication Year Start, indicating the year the journal started publication.

### Analysis of the journals

**Figure 6:**
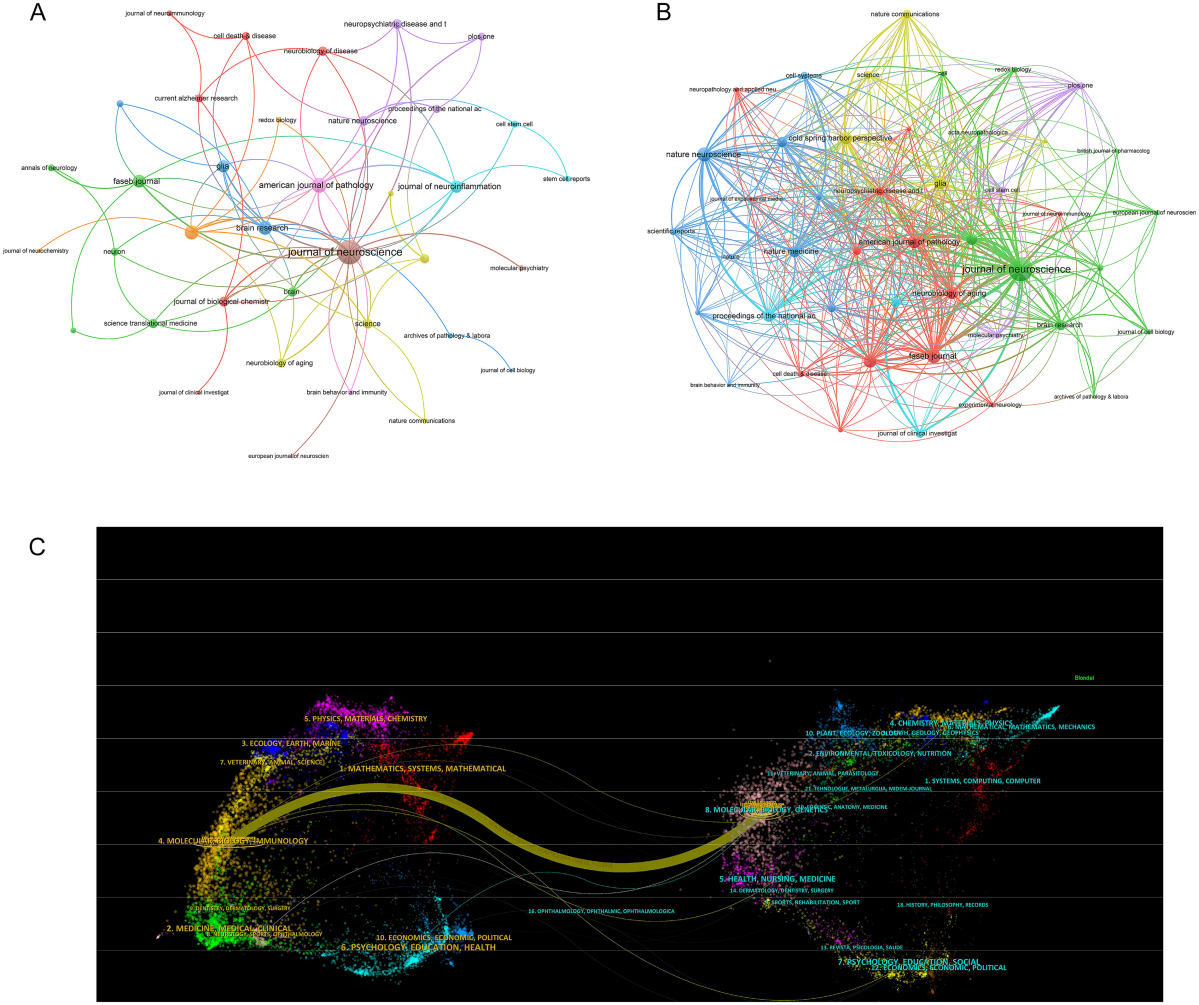
Analysis of journal. **(A)** Visualization networks of journal collaborations. The frequency with which journals are cited together within the same articles reflects thematic or topical connections between the research they publish. **(B)** Visualization coupling networks of journal collaborations. The extent to which journals are linked is based on common references cited in their articles, indicating a shared intellectual foundation or research focus. **(C)** A dual-map overlay of journals related to research on astrocytes in AD. Citation paths at a disciplinary level were demonstrated in a dual-map overlay. The left of the map represented the cite journals and the right of the map represented the cited journals. Citation trajectories are colored based on the citing regions. The width of the paths is proportional to the z-score-scaled citation frequency.

The top 20 journals considering the research number of astrocytes and AD in Table 4, include the impact indicators, such as h-index, g-index, m-index, and impact factor (IF). The *Journal of Neuroscience* contributed the most publications (15) with an h-index of 15 (Q1, IF = 4.4), followed by *Nature Neuroscience* (7) with an h-index of 7 (Q1, IF = 21.2). For total citations, the *Journal of Neuroscience* had the most total citations (374), followed by *Proceedings of the National Academy of Sciences of the United States of America* (270) and *Journal of Biological Chemistry* (255). Among the 45 journals with at least 1 occurrence, the three key journals with the highest total link strength in co-occurrence networks were *the Journal of Neuroscience* (total link strength = 36), *American Journal of Pathology* (total link strength = 14), *and Brain Research* (total link strength = 12) (Figure 6A). Besides, the three key journals with the highest total link strength in coupling networks were *Journal of Neuroscience* (total link strength = 600), *Nature Neuroscience* (total link strength = 209), and *FASEB Journal* (total link strength = 188) (Figure 6B). The dual map of this journal showed one main citation path (Figure 6C). It is worth noting that publications from molecular/biology/immunology journals were mainly cited by molecular/biology/genetics journals in the context of astrocytes in AD research.

**Table 4:** Bibliometric indicators of high-impact journals.

Journal	h_index	g-index	m-index	TP	TP_rank	TC	TC_rank	PY_start	IF_2023	JCR_2023
Journal of Neuroscience	15	15	0.577	15	1	374	1	2000	4.4	Q1
Nature Neuroscience	7	7	0.636	7	2	94	14	2015	21.2	Q1
Glia	5	5	0.200	5	3	119	9	2001	5.4	Q1
Journal of Biological Chemistry	5	5	0.192	5	4	255	3	2000	4	Q2
Neurobiology of Aging	4	4	0.250	4	5	162	6	2010	3.7	Q2
Proceedings of the National Academy of Sciences of the United States of America	4	4	0.235	4	6	270	2	2009	9.4	Q1
American Journal of Pathology	3	3	0.120	3	7	100	11	2001	4.7	Q1
Brain Research	3	3	0.120	3	8	95	13	2001	2.7	Q3
FASEB Journal	3	3	0.188	3	9	44	33	2010	4.4	Q2
Journal of Neuroinflammation	3	3	0.167	3	10	53	29	2008	9.3	Q1
Nature Medicine	3	3	0.250	3	11	92	15	2014	58.7	Q1
Neuron	3	3	0.125	3	12	179	5	2002	14.7	Q1
Brain	2	2	0.200	2	13	55	28	2016	10.6	Q1
Cell Stem Cell	2	2	0.154	2	14	14	79	2013	19.8	Q1
Cold Spring Harbor Perspectives in Biology	2	2	0.182	2	15	4	207	2015	6.9	Q1
Journal of Neurochemistry	2	2	0.133	2	16	140	8	2011	4.2	Q2
Nature	2	2	0.286	2	17	203	4	2019	50.5	Q1
Nature Communications	2	2	0.167	2	18	26	48	2014	14.7	Q1
Neuropathology and Applied Neurobiology	2	2	0.095	2	19	16	70	2005	4	Q1
Science	2	2	0.118	2	20	152	7	2009	44.7	Q1

h_index, The h-index of the journal, which measures both the productivity and citation impact of the publications; g_index, The g-index of the journal, which gives more weight to highly-cited articles; m_index, The m-index of the journal, which is the h-index divided by the number of years since the first published paper; IF_2023, Impact Factor in 2023, indicating the average number of citations to recent articles published in the journal; JCR_2023, The quartile ranking of the journal in the Journal Citation Reports in 2023, indicating the journal’s ranking relative to others in the same field (Q1: top 25%, Q2: 25–50%, Q3: 50–75%, Q4: bottom 25%). TP, Total Publications; TP_rank, Rank of Total Publications; TC, Total Citations; TC_rank, Rank of Total Citations; PY_start, Publication Year Start, indicating the year the journal started publication.

### Analysis of the references

**Figure 7:**
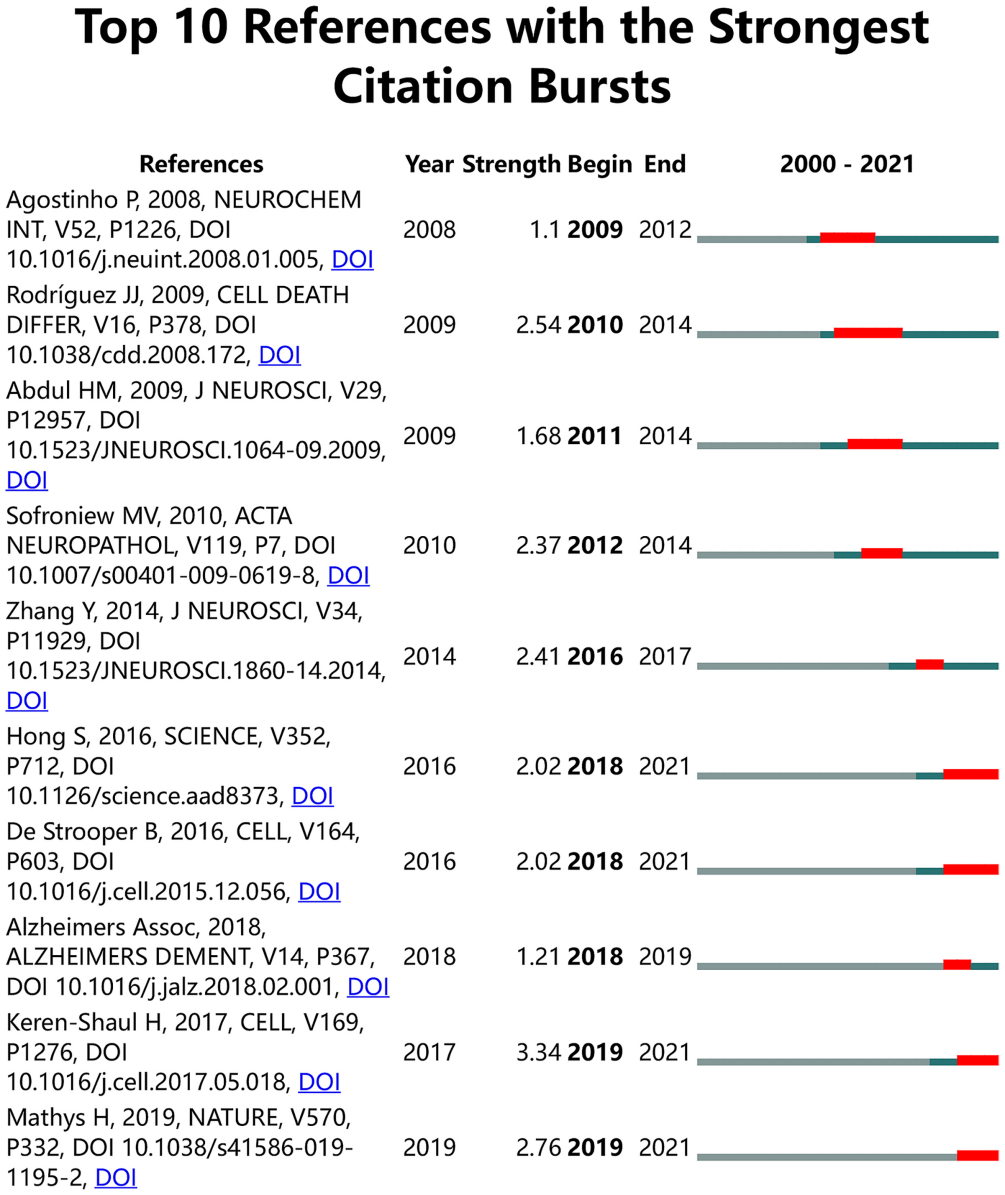
Top 10 references with strongest citation burst. The blue lines represent the period, and the red lines indicate the burst periods of the references.

Top 10 references with the most pronounced citation bursts were shown in Figure 7. The majority of these references witnessed citation bursts after 2010. Notably, none of these references maintained sustained influence for more than 5 years after their citation burst. Only one reference, published in 2009, remained highly influential as of 2014. Among the top 10 references, published in *Cell* in 2017 Keren-Shaul et al. (2017), held the top position in terms of citation burst value (strength = 3.34).

### Analysis of the keywords

**Figure 8:**
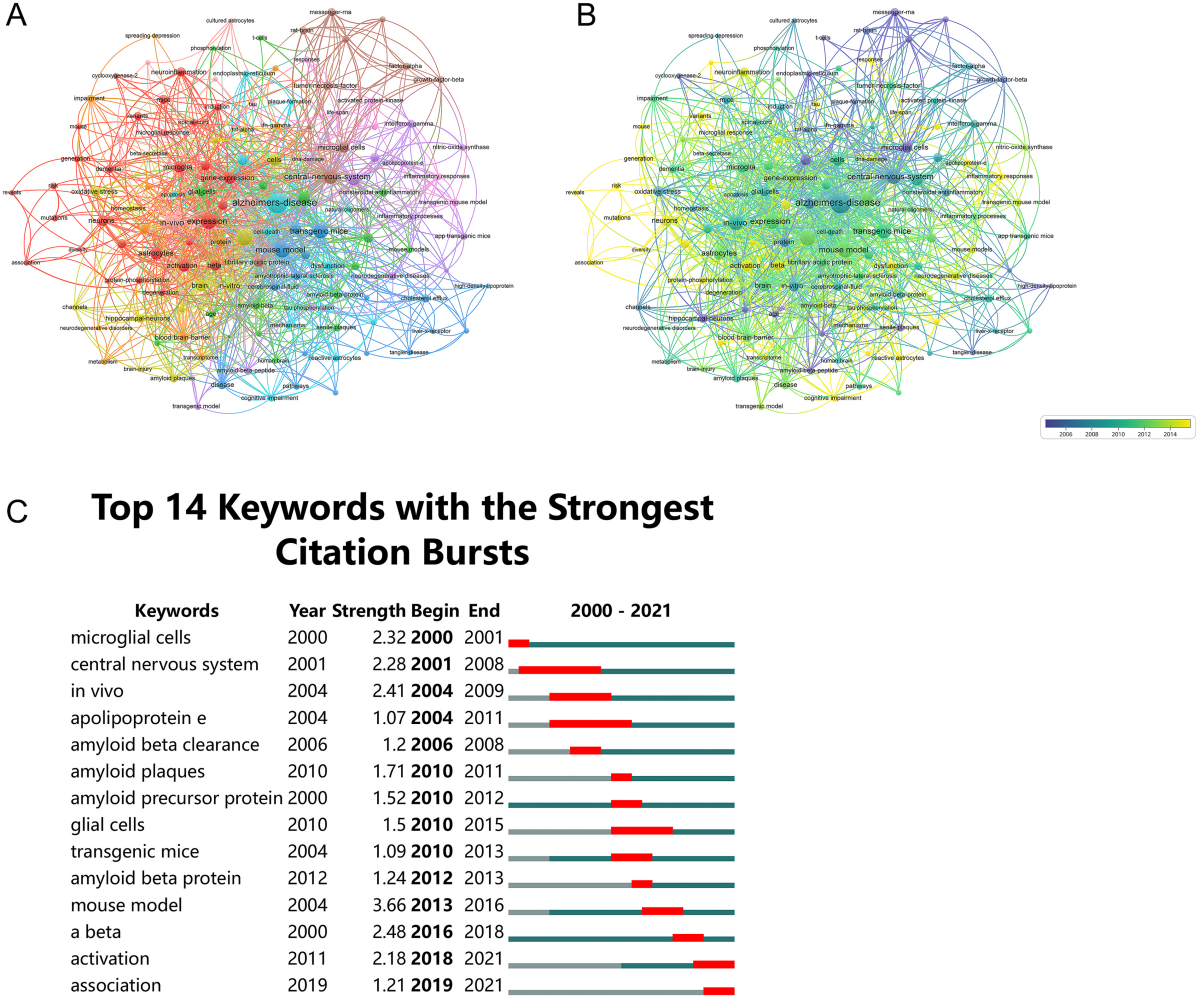
Analysis of keywords. **(A)** Keyword co-occurrence network. This network visualization displays the co-occurrence of keywords in selected literature. Each node represents a keyword, with size indicating its frequency of occurrence. Links between nodes represent co-occurrence in the same documents, with thicker lines showing stronger associations. Colors indicate different research clusters. Total link strength measures the frequency of co-authorship between keywords. **(B)** Time-overlapping co-occurrence analysis network of keywords. This network visualization displays the co-occurrence of keywords in selected literature. Each node represents a keyword, with size indicating its frequency of occurrence. Links between nodes represent co-occurrence in the same documents, with thicker lines showing stronger associations. Colors reflect the average publication year of the articles, as indicated by the color gradient at the bottom right. The transition from purple to green to yellow represents the timeline of keywords, with purple indicating older terms and yellow representing the most recent ones. **(C)** Top 14 keywords with the strongest citation burst. The blue lines represent the duration of the keyword, and the red lines indicate the burst periods of the keywords.

The analysis of keywords offered insights into the research hotspots and trends in the field. The top 20 keywords with the highest occurrences were shown in Table 5, including “mouse model” (15 occurrences and total link strength = 78), “Aβ” (15 occurrences and total link strength = 76), and “expression” (15 occurrences and total link strength = 73). The keyword co-occurrence network was depicted in Figure 8A. Finally, six possible study directions were identified, which are as follows: (1) Red cluster: astrocytes-induced neuroinflammation in AD; (2) Yellow cluster: oxidative stress; (3) Blue cluster: mouse model in AD exploration; (4) Brown cluster: molecular pathogenesis involved in astrocytes and AD; (5) Green cluster: role of Aβ in astrocytes and AD; (6) Purple cluster: biomarkers of AD via astrocytes.

**Table 5:** Top 20 keyword co-occurrence network analysis.

Id	Keyword	Occurrences	Total link strength
28	alzheimers-disease	26	128
351	mouse model	15	78
3	a-beta	15	76
201	expression	15	73
538	transgenic mice	15	69
265	in-vivo	15	68
111	central-nervous-system	13	66
37	amyloid precursor protein	8	46
61	astrocytes	9	46
329	microglial activation	7	45
109	cells	8	42
13	activation	8	40
90	brain	7	39
222	gene-expression	9	38
168	deposition	7	37
327	microglia	9	37
71	beta	6	33
264	in-vitro	6	33
330	microglial cells	6	33
381	neurons	7	33

The time-overlay visualization map was established in Figure 8B. Earlier studies, indicated by darker nodes, primarily explored broader themes such as “messenger RNA,” and “IFN-*γ*,” emphasizing the pathogenesis of AD. Meanwhile, recent studies have concentrated on topics such as “mechanisms,” and “activation,” highlighting the importance of the mechanism of AD via astrocytes for precise prevention and treatment.

In Figure 8C, the intensity of the top 14 keywords with the most notable bursts ranged between 1.07 and 3.66, reflecting varying degrees of impact and attention within the research community. Prominently, the keywords “central nervous system” (2001–2008) and “Apolipoprotein E” (2004–2011) exhibited the most lasting bursts and “mouse model” had the highest burst strength of 3.66. The earliest bursting keywords were “central nervous system,” “*in vivo*,” and “Apolipoprotein E,” while the most recent bursting keywords comprised “Aβ,” “activation” and “association” since 2016.

## Discussion

AD represents a significant global health challenge characterized by a progressive deterioration of cognitive functions, memory impairment, and, ultimately, the loss of the ability to perform daily activities independently (Soria Lopez et al., 2019). Astrocytes have recently attracted significant attention for their potential involvement in the pathogenesis of AD. These glial cells are essential for maintaining neuronal health, regulating the integrity of the blood–brain barrier, and modulating synaptic activity (Patani et al., 2023). Astrocytes can release neurotrophic factors to support neuron survival or pro-inflammatory cytokines that lead to neuroinflammation and synaptic dysfunction (Patani et al., 2023). This dual functionality of astrocytes in AD implies that targeting their activity may represent a viable therapeutic strategy (An et al., 2024). Nonetheless, the exact mechanisms through which astrocytes affect the progression of AD remain inadequately elucidated, thereby warranting comprehensive investigation into their multifaceted roles. This study used bibliometric methods to assess the literature on astrocytes and AD, elucidating the research overview, hotspots, and prospective directions within this domain. These studies examining the relationship between AD and astrocytes may be driven both by a deepening understanding of the pathogenesis of AD and by the continued emergence of new technologies and approaches, as well as the development of potential therapeutic targets and drugs (Pelkmans et al., 2024; Citron, 2010). This indicates that interest in this area of research remains strong, and the number of publications is expected to continue to grow in the future.

## General information

The number of publications is an important indicator for assessing the scientific capacity of a country, organization, or individual. The findings of this study indicated that the United States held the top position in terms of publication and citation volume, underscoring the emphasis placed on research. The reason may be the severe condition of AD in the United States. Specifically, an estimated 6.9 million Americans aged 65 and older are living with AD today. This number could grow to 13.8 million by 2060, barring the development of medical breakthroughs to prevent or cure AD. Official AD death certificates recorded 119,399 deaths from AD in 2021, which remains the fifth-leading cause of death among Americans aged 65 and older (Alzheimer’s Association Report, 2024). Therefore, the United States has introduced several policies related to AD. For example, in 2012, the USA launched the National Plan to Combat Alzheimer’s Disease and set goals for treating and preventing AD and related dementias by 2025 (Pollán et al., 2003). WUSTL from the United States had the highest number of publications. Besides, all top 10 institutions came from the United States, further suggesting that the USA has a great influence in this research field and is in the absolute leading position.

In accordance with analysis of the authors, Holtzman David M had the most papers and citations, indicating his greatest influence and most outstanding contributions to the field of astrocytes in AD. Holtzman David M from WUSTL, showed, in part, how ApoE4 contributes to AD (Parhizkar and Holtzman, 2022), development of cerebrospinal fluid (CSF) biomarkers for AD (Perrin et al., 2011), demonstration of how synaptic/neuronal activity and sleep affect Aβ and tau levels dynamically *in vivo* acutely and chronically (Wang and Holtzman, 2020), determined that ApoE4 and triggering receptor expressed on myeloid cells 2 (TREM2) contribute to the brain’s innate immune response that influences amyloid-induced tau seeding and spreading (Shi and Holtzman, 2018), as well as tau-mediated neurodegeneration (Shi et al., 2017).

The leading journals in the field, including *Journal of Neuroscience* and *Nature Neuroscience*, are ranked high in publication volume, providing an appropriate platform for disseminating significant research findings. Given their impact and the dense citation network revealed in the analysis, these journals represent key outlets for researchers aiming to contribute to the field or stay updated on emerging trends. The number of citations can be one of the indicators of the scholarly impact of a publication. Highly cited publications tend to represent the underlying themes of a field of study. The most cited article published by Moloney AM et al. in 2010 elucidates that insulin-like growth factor-1 receptor (IGF-1R) and insulin receptor (IR) signaling are compromised in AD neurons and suggests that neurons that degenerate in AD may be resistant to IGF-1R/IR signaling (Moloney et al., 2010). The second most cited article by Guo ZY et al. in 2014 posits that direct reprogramming of reactive glial cells into functional neurons in vivo could provide an alternative approach for the repair of injured or diseased brain (Guo et al., 2014). The third most cited article by Zhou YY in 2020 demonstrated that variants of the TREM2 increase AD risk in mouse models of AD (Zhou et al., 2020).

## Hotspots and frontiers

Keywords provide a critical lens to uncover shifting research priorities and emerging themes. The co-occurrence network revealed four major clusters

each representing different dimensions of this field as follows:

### Cluster 1 (red): astrocytes-induced neuroinflammation in AD

Neuroinflammation generally refers to an inflammatory response within the CNS that can be caused by various pathological insults, including infection, trauma, ischaemia, and toxins, marked by the release of pro-inflammatory factors, including IL-1β, IL-6, IL-18, and etc. (Mangalmurti and Lukens, 2022). The innate immune cells involved in this process are primarily microglia and astrocytes (Farhy-Tselnicker and Allen, 2018). Astrocytes are acknowledged to respond to pathological insults (including mechanical injury, ischaemia, and abnormal protein aggregates) through reactive gliosis, which is part of the neuroinflammatory process (Pekny et al., 2014). Furthermore, excessive production of neurotoxic factors modulates astrocytes’ amyloid precursor protein (APP) processing homeostasis, which leads to increased Aβ load and toxicity (Singh, 2022). These cascade reactions contribute to the pathogenesis of neurodegenerative diseases, such as AD (Spittau, 2017).

### Cluster 2 (yellow): oxidative stress

Oxidative stress develops due to an imbalance between the production of free radicals and antioxidants in the mitochondria (Sies et al., 2017). In AD, oxidative stress appears secondary to mitochondrial dysfunction, which may lead to synaptic Aβ-induced damage (Dhapola et al., 2024). This has been linked to increased levels of reactive oxygen species (ROS) and reactive nitrogen species (RNS). Under physiological conditions, astrocytes are fundamental for neuronal antioxidant production, since they synthesize and deliver amino acids such as glycine and cysteine for glutathione (GSH) production in neurons (Rodríguez-Giraldo et al., 2022). However, during AD, Aβ levels have been shown to be directly correlated with ROS production, with large amounts of ROS inducing a neurotoxic profile in astrocytes through the expression of inducible nitric oxide synthase (iNOS), causing nitrosative stress and toxic nitration in neurons(Chun and Lee, 2018). The process of astrocytic iNOS stimulation due to Aβ has been shown to be dependent on IL-1β and tumor necrosis factor (TNF), through a nuclear factor kappa-B (NF-κB) inducing kinase (NIK)-dependent signaling mechanism (Rodríguez-Giraldo et al., 2022). These findings suggest that the induction of neurotoxic versus neuroprotective RA profiles is correlated with the level of ROS production, forming a continuous cycle between neuroinflammation and oxidative stress.

### Cluster 3 (blue): mouse model

Animal models serve as an indispensable tool to understand the molecular and cellular mechanisms underlying AD pathogenesis, to evaluate the specific therapeutic approaches, and to discover translatable biomarkers for AD diagnosis (Pádua et al., 2024). In general, most of the commonly used AD mice were developed by overexpression of familial AD (FAD)-related APP mutations, either alone or in combination with presenilin 1 (PSEN1) and PSEN2 mutations (Qian et al., 2024). The advantages of using mouse models for AD research include the following: (i) a relatively large genome similar to that of humans; (ii) cost-effective for large-scale, high-throughput, and long-term investigation related to AD progression, intervention, and treatment; (iii) well established strategies for genetic manipulation in AD mice; and (iv) well established cognitive behavioral analysis approaches(Qian et al., 2024).

### Cluster 4 (brown): molecular pathogenesis

Excessive activation of astrocytes, in conjunction with other changes, including Aβ and tau accumulation, exacerbates neurodegeneration in AD (Wu et al., 2023). The role of astrocytes in neurogenesis, synaptogenesis, angiogenesis, and axonal remodeling in other brain diseases suggests astrocytes as potential therapeutic targets (Liu and Chopp, 2016). The role of astrocytes in AD initiation and progression is further underscored by changes in astrocytes-associated/secreted cytokines. For instance, ApoE4, primarily expressed by astrocytes in the brain, is a multifunctional protein crucial for lipid metabolism and neurobiology, such as synapse formation and maintenance, and the progression of AD (Koutsodendris et al., 2022). Furthermore, another astrocytes-associated chemokine, monocyte chemoattractant protein-1 (MCP-1), also contributes to AD pathology (Singh et al., 2021). In a two-year follow-up study, plasma MCP-1 levels were significantly higher in AD patients than in healthy controls (He et al., 2016). Notably, these factors may be potential biomarkers in AD.

### Cluster 5 (purple): astrocytes as biomarkers in AD

Activated astrocytes may serve as *in vivo* fluid biomarkers of AD. Pronounced alterations in the astrocytic expression of glial fibrillary acidic protein (GFAP), S100B, and chitinase-3-like protein 1 (YKL-40) have been identified in patients with AD. Clinical corollaries are emerging; for instance, it is recognized that an AD phenotype with blood GFAP elevation confers more rapid cognitive decline (Holper et al., 2024). Similarly, another meta-analysis further demonstrated that GFAP and YKL-40 levels in the cerebrospinal fluid and S100B levels in the blood were found to be significantly increased in patients with AD (Bellaver et al., 2021). Thus, it is important to understand astrocytes networks on a global level. Both functional and structural connectivity mapping studies are needed to establish how astrocytes interact with neurons, other glial cells, and immune cells in health and disease (Linnerbauer et al., 2020). Thus, these biomarkers can be potential therapeutic targets for AD.

### Cluster 6 (green): aβ protein

Aβ pathology is associated with astrocytes reactivity, and GFAP levels, as a biomarker of reactive astrocytes, mainly reflect a response to Aβ pathology (Ferrari-Souza et al., 2022). Previous *post-mortem* observations also supported that reactive astrocytes overexpressing GFAP are found in the vicinity of Aβ plaques (Serrano-Pozo et al., 2011). Furthermore, it was reported that the topography of GFAP-immunopositive astrocytes resembles the distribution of Aβ plaques in AD (Ferrari-Souza et al., 2022). Wang et al. proposed that astrocytic cholesterol is a key regulator of neuronal Aβ accumulation. Specifically, treatment with cholesterol-free ApoE4 or knockdown of cholesterol synthesis in astrocytes decreases cholesterol levels in cultured neurons and causes APP to traffic out of lipid clusters, where it interacts with *α*-secretase and gives rise to soluble APP-α, a neuronal protective product of APP(Wang et al., 2021).

The analysis of keywords serves as a valuable tool for identifying research hotspots and trends. The utilization of literature keyword occurrence and clustering can unveil the underlying research structure in the field of astrocytes in AD. Earlier studies primarily explored broader themes such as “messenger RNA,” and “IFN-*γ*,” which emphasize the pathogenesis of AD. A number of studies have identified several mRNAs, such as miR-501-3p and miR-223, that are significantly differentially expressed in the blood from patients with AD compared with normal control samples, indicating their key functions in the pathogenesis of AD (Wang et al., 2020), providing novel insights into the molecular mechanisms underlying AD. Besides, these results suggested the hotspot of epigenetic modifications in astrocytes in AD. Epigenetic studies in neurodegenerative diseases provide evidence that genetic and non-genetic factors alter gene expression profiles in neurons and astrocytes through aberrant epigenetic mechanisms. For example, DNA methylation and histone marks at promoters contribute to transcriptional dysregulation of genes that are directly implicated in AD pathogenesis, neuroplasticity, cognition, and astrocytes activation (van Zundert and Montecino, 2025). Astrocytes are intimately involved in immunological and inflammatory events occurring in the CNS, due to their ability to secrete and respond to a large number of immunoregulatory cytokines/chemokines such as IL-1β, IL-6, and interferon-gamma (IFN-γ), etc. (Qin and Benveniste, 2012). These cytokines can further contribute to development of AD (Qin and Benveniste, 2012). Among them, IFN-γ is pivotal for driving Toll-like receptor (TLR)-activated microglia into neurotoxic phenotypes that induce metabolic and oxidative stress, severe neural network dysfunction, and neuronal cell death. These lines of evidence suggest that IFN-γ may act as a “master control” of peripheral (adaptive) immune cells in microglia-mediated inflammatory neurodegeneration as well as during host inflammatory attack. Moreover, these features occur to a variable degree in AD (Kann et al., 2022). Recent studies have concentrated on topics such as “mechanisms,” and “activation,” highlighting the importance of the mechanisms of AD via astrocytes for precise prevention and treatment.

Consistently, the keyword citation burst analysis also revealed several terms with significant occurrences since 2016, including “Aβ,” “activation” and “association.” There is a bidirectional interaction between astrocytes and Aβ. Aβ is mainly produced by neurons and cleared by immune cells such as lymphocytes and phagocytes to maintain normal levels of Aβ in brain tissue, while pathogenesis of AD has been attributed to extracellular aggregates of Aβ plaques (Tiwari et al., 2019). If there is chronic inflammation of neurons, the astrocytes and other inflammatory cells become hyperactivated, which can promote the expression of Aβ, neuronal fibrillary tangles, and Aβ peptide deposits (Tuppo and Arias, 2005). When large amounts of Aβ are present around astrocytes, neuronal mortality around astrocytes increases via promoting neuronal apoptosis (Kaur et al., 2019). Thus, mild activation of astrocytes is beneficial as it clears the cell debris, damaged neurons, and Aβ (Dhapola et al., 2021). How to enhance the astrocytes-mediated Aβ clearance is a potential therapeutic strategy as well as research trend. Bilobalide promotes the expression of Aβ-degrading enzymes, including Neprilysin (NEP), insulin-degrading enzyme (IDE), and matrix metallopeptidase 2 (MMP2), in astrocytes to facilitate astrocyte-mediated Aβ clearance, thereby rescuing neuronal deficiency (Xiang et al., 2021). Besides, previous studies underscore the role of Rho GTPases—particularly RhoA, Rac1, and Cdc42—in regulating Aβ clearance and neuroinflammation. Targeting Rho GTPase signaling pathways in astrocytes may offer a promising therapeutic approach to mitigate neuroinflammation, enhance Aβ clearance, and slow disease progression, ultimately improving cognitive outcomes in AD patients (Park et al., 2024). Anti-inflammatory molecules like minocycline are also employed to reduce Aβ and tau pathologies by mitigating the release of pro-inflammatory cytokines from glial cells (Dhapola et al., 2021). Additionally, the mitophagy process is considered quite helpful in reducing inflammation due to glial cells as it promotes the phagocytosis of overactivated glial cells (Dhapola et al., 2021). Enhancement of astrocytic autophagic plasticity also accelerates the Aβ clearance and maintains cognitive function (Kim et al., 2024). These studies highlight the astrocytes-based therapeutic approaches, which provides promise to translate into clinical applications or inform future therapeutic directions.

Promising results from preclinical studies have led to ongoing human clinical trials. A randomized, phase 1b/2 trial primarily demonstrated pepinemab, a high affinity, semaphorin 4D (SEMA4D) blocking antibody, can prevent astrocyte activation and reduce brain inflammation (Siemers et al., 2024). A Phase 1 trial confirmed the safety, tolerability, and feasibility of senolytic therapy against astrocytes senescence in patients with mild AD through a combination of oral dasatinib and quercetin (Gonzales et al., 2023). However, the trials on astrocytes-based therapeutic approaches are still lack. Thus, more trials should be further conducted in the future.

Based on the important role of Aβ in AD, promoting Aβ clearance, reducing inflammation, or repairing the normal physiological function of astrocytes play important roles in the treatment and prevention of AD in the future. Moreover, an increasing amount of research funding should be allocated to this field to support in-depth studies on the pathogenesis of astrocytes in AD, which will facilitate further research on new therapeutic targets or predictive factors for early intervention. Moreover, more corresponding double-blind randomized controlled trials should also be conducted to verify the efficacy and safety of these identified targets, which also emphasizes that healthcare policy frameworks should prioritize resource allocation to this therapeutic domain, with strategic emphasis on addressing unmet clinical needs through targeted funding mechanisms and regulatory pathway optimization.

## Strengths and limitations

This study employed the bibliometric approach for visualizing the research on astrocytes and AD, thereby gaining a better understanding of the hotspots and trends in this field. However, it is important to acknowledge certain constraints within this study. Firstly, this study included only English-language publications, which may have led to language bias and the omission of relevant studies published in other languages. Secondly, the analysis was based solely on data retrieved from the WoSCC. While WoSCC is widely used in bibliometric research, relying on a single database may limit the scope and comprehensiveness of the findings. Integrating data from other major databases such as PubMed or Scopus could provide a more complete overview of the research landscape. Thirdly, as this is a rapidly evolving research field, recently published studies may be underrepresented in the analysis due to their limited citation accumulation at the time of data collection, even if they were published in high-quality journals. Additionally, bibliometric methods inherently rely on the frequency of terms and citation patterns, which may limit their ability to capture emerging or less-established topics. As a result, some recent developments may not be fully reflected in our findings. Finally, keyword analysis may not provide enough information to reveal deeper research motivations and specific research processes.

## Conclusion

This is the first bibliometric analysis of the top 100 cited research on astrocytes and AD from 2000 to 2025, including the number and impact of research findings, research hotspots, and future trends. The United States published the most articles in the field of astrocytes in AD. WUSTL has published the most papers of all institutions. Holtzman David M was the most influential author with the most articles and the highest m-index. *Journal of Neuroscience* is the most active journal. Keyword analysis indicated the growing interest in the pathogenesis of astrocytes in AD. Future studies on the mechanisms underlying astrocytes in AD will facilitate further research on new therapeutic approaches.

## Data Availability

The original contributions presented in the study are included in the article/supplementary material, further inquiries can be directed to the corresponding author.
